# Brilliant iridescence of *Morpho* butterfly wing scales is due to both a thin film lower lamina and a multilayered upper lamina

**DOI:** 10.1007/s00359-016-1084-1

**Published:** 2016-04-12

**Authors:** M. A. Giraldo, D. G. Stavenga

**Affiliations:** Biophysics Group, Institute of Physics, University of Antioquia, Calle 70 #52-21, AA 1226, Medellín, Colombia; Computational Physics, University of Groningen, Nijenborgh 4, 9747 AG Groningen, The Netherlands

**Keywords:** Microspectrophotometry, Scatterometry, Animal coloration, Nymphalidae, Melanin

## Abstract

Butterflies belonging to the nymphalid subfamily, Morphinae, are famous for their brilliant blue wing coloration and iridescence. These striking optical phenomena are commonly explained as to originate from multilayer reflections by the ridges of the wing scales. Because the lower lamina of the scales of related nymphalid butterflies, the Nymphalinae, plays a dominant role in the wing coloration, by acting as a thin film reflector, we investigated single blue scales of three characteristic *Morpho* species: *M. epistrophus, M. helenor* and *M. cypris*. The experimental data obtained by spectrophotometry, scatterometry and scanning electron microscopy demonstrated that also in the *Morpho* genus the lower lamina of both the cover and ground scales acts as an optical thin film reflector, contributing importantly to the blue structural coloration of the wings. Melanin pigment has a contrast-enhancing function in a sub-class of ground scales.

**Electronic supplementary material:**

The online version of this article (doi:10.1007/s00359-016-1084-1) contains supplementary material, which is available to authorized users.

## Introduction

Butterflies are generally recognized as the order of insects with a most diverse patterning and coloration. The evolution of the color patterns has been driven by different causes, such as sexual selection, conspecific recognition, camouflage, and mimicry. The colors are due to the lattice of scales that cover both sides of the wing substrate and are created by the interaction of light with the scales’ optical materials (Srinivasarao 1999; Kinoshita et al. 2008). Depending on the structure and/or pigmentation of the scales, different wing areas can have distinct colors (Yoshioka and Kinoshita 2006; Stavenga et al. 2014).

Butterfly wing scales generally have a very asymmetric structure and basically consist of two chitin layers. One layer is generally a smooth, more or less flat plate, which faces the wing substrate. It, therefore, is called the adwing or lower lamina. The other layer consists of an array of ridges running parallel to the scale’s longitudinal axis, connected by crossribs, thus together forming a bidimensional grating (Ghiradella 1998). This layer is called the abwing or upper lamina. The two layers are connected by pillar-like trabeculae. The areas delimited by the ridges and crossribs, the windows, are called to be closed when containing a membrane, while open windows have no membrane (Ghiradella 2010). Within the ridges partly overlapping lamellae can be commonly distinguished. With sufficient overlap, the lamellae create a multilayer, resulting in structural coloration. This is especially the case in several butterfly species belonging to the genus *Morpho*, which have large wing areas covered by wing scales with most elaborate ridges, consisting of large stacks of lamellae, acting as brightly reflecting multilayers (Kinoshita et al. 2002a; Vukusic and Sambles 2003). Characteristically, transmission electron micrographs of the wing scales show a Christmas-tree-like structure (Lippert and Gentil 1959; Ghiradella 1984). Similar structures are encountered in other nymphalid subfamilies, for instance the Apaturinae (Ćurčić et al. 2012), but also in other lepidopteran families as the Lycaenidae (Tilley and Eliot 2002); all butterfly wing scales with multilayered ridges are referred to as *Morpho* type (Ghiradella et al. 1972; Tilley and Eliot 2002; Giraldo et al. 2008).

Scales which contain wavelength-selective absorbing pigments exhibit pigmentary coloration. Pigmentary and structural coloration are frequently combined. For instance, scales at the dorsal forewings of the Purple Tip butterfly *Colotisregina* produce a distinct blue iridescence, due to *Morpho*-type multilayers, but they also contain pigment granules that absorb in the short-wavelength range and scatter long-wavelength light. The blue structural and red pigmentary coloration together cause a characteristic purple color (Giraldo et al. 2008).

Many Morphinae, with their stunning blue iridescence and rather large wing size, have become the iconic examples of brilliant, eye-catching butterflies (Mason 1923; Kinoshita et al. 2002b, 2008; Kinoshita and Yoshioka 2005). Numerous papers have studied the multilayer optics of the scale ridges, implicitly assuming that the reflection properties of *Morpho* scales are determined by the ridges (Gralak et al. 2001; Kinoshita et al. 2008), neglecting the possibly important contribution of the lower lamina. A notable exceptional case is that of *Morpho didius* cover scales, where the lower lamina was recognized to have a blue color (Yoshioka and Kinoshita 2004).

Recently, studies on a number of papilionid and nymphaline species indicated that the lower lamina acting as a thin film may generally play a primary role in scale coloration (Trzeciak et al. 2012; Wilts et al. 2012; Stavenga 2014). Actually, several *Morpho* species do not exhibit the strong blue iridescence of their famous relatives, and so apparently not all species have the prominently reflecting scales. The phylogenetic proximity of the Morphinae and Nymphalinae subfamilies therefore suggested to take a fresh look into a possible contribution of the scales’ lower lamina.

Here, we report the optical and morphological properties of single blue scales belonging to three exemplary species of Morphinae. Starting from the simplest scale type of *Morpho epistrophus* we compared its optics with that of the more complex scales of *M. helenor* and *M. cypris*. We demonstrate that, like in other butterfly species, also in *Morpho* butterflies the scales’ lower lamina, acting as an optical thin film, importantly contributes to scale coloration.

## Materials and methods

### Animals and light microscopy

Specimens of *M. epistrophus*, *M. helenor* and *M. cypris* were purchased from commercial suppliers. Single wing scales were obtained by gently pressing the wings to a glass microscope slide. An isolated scale was then glued to the tip of a glass micropipette, which was mounted on the rotatable stage of a Zeiss Universal Microscope (Zeiss, Oberkochen, Germany). Bright- and dark-field photographs were made using a Zeiss Epiplan 16×/0.35 and 8×/0.2 objective, respectively, and a Kappa DX-40 (Kappa Optronics GmbH, Gleichen, Germany) digital camera.

### Imaging scatterometry

To investigate the spatial reflection characteristics of the scales, we performed imaging scatterometry. A single scale attached to the tip of a glass micropipette was positioned at the first focal point of the ellipsoidal mirror of the imaging scatterometer. A white light beam with a narrow aperture (<5º) was focused onto a small circular area (diameter ~ 30 µm) of the isolated scale, and the spatial distribution of the far-field scattered light was then recorded with a digital camera (for details, see Stavenga et al. 2009). Scatterograms thus represent the reflection hemisphere (e.g. Fig. 2c). The red circles in the scatterograms indicate angles of 5º, 30º, 60º and 90º.

### Scanning electron microscopy (SEM)

Scanning electron microscopy was performed on single scales placed on a carbon stub, with either the upper or lower lamina exposed. To reveal the transversal morphology of the scales, scales were trans-sectioned with a razor blade. Prior to imaging, the samples were sputtered with gold.

### Spectrophotometry

Reflectance spectra of both scale sides were made with a home-built microspectrophotometer (MSP), which consists of a Leitz Ortholux epi-illumination microscope connected to a fiber optic spectrometer AvaSpec-2048 (Avantes, Eerbeek, The Netherlands). The light source was a xenon arc, and the microscope objective was an Olympus LUCPlanFL N 20×/0.45 (Olympus, Tokyo, Japan); a white reflectance standard (WS-2, Avantes) served as a reference. Due to the glass optics, the MSP spectra were limited to wavelengths >350 nm.

We approximated the reflectance spectra measured from the adwing side with the reflectance spectrum of a thin film, normally illuminated, using the classical Airy formula (e.g. Stavenga 2014). For the refractive index we used the Cauchy equation *n*(*λ*) = *A* + *Bλ*^−2^, with *A* = 1.517 and *B* = 8.80 × 10^3^ nm^2^, determined from glass scales of the butterfly *Graphium sarpedon* (Leertouwer et al. 2011). A small offset was added to the calculated spectra to account for the scattering by the structures of the upper lamina.

The reflectance tile used as reference was a perfect diffuser while the investigated scales were partly specular. Because of the limited aperture of the microspectrophotometer distinctly overestimated reflectance values were thus obtained. The thin film calculations showed that the measured adwing spectra were too large by about a factor 4. The experimental reflectance spectra shown in Figs. 2, 3 and 4 were therefore divided by 4, so becoming comparable with the calculated spectra. In some cases the lower lamina was found to contain melanin. In the thin film calculations, the lower lamina’s refractive index was then assumed to have an imaginary part of 2.5exp(−*λ*/270) (see Fig. S1; Stavenga et al. 2015).

## Results

**Fig. 1:**
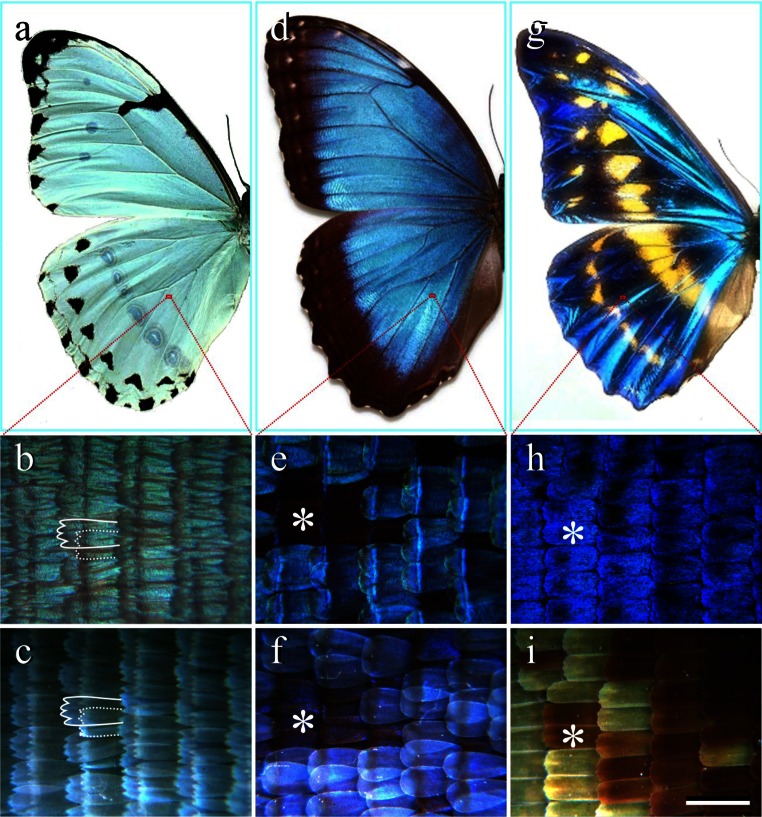
Three exemplary *Morpho* butterflies, *M. epistrophus* (**a**–**c**), *M. helenor* (**d**–**f**), and *M. cypris* (**g**–**i**). **a**, **d**, **g** Photographs of the *left* wings. **b**, **e**, **h** Bright-field micrographs of a small area indicated by the *red lines*. **c**, **f**, **i** Dark-field micrographs of the same areas. In the micrographs of *M. epistrophus* (**b**, **c**) a cover and ground scale are indicated by a *solid* and *dashed line*, respectively. The *asterisks* in the micrographs of *M. helenor* (**e**, **f**) indicate an area where the cover scales were removed; cover scales are unpigmented and have a rounded tip. The *asterisks* in the micrographs of *M. cypris* (**h,** **i**) indicate an iridescent, pigmented scale. *Bar* **i** 200 µm

The investigated *Morpho* butterflies, *M. epistrophus*, *M. helenor* and *M. cypris*, have wings with bluish colors varying in saturation and patterning (Fig. 1a, d, g). Applying both bright-field (Fig. 1b, e, h) and dark-field (Fig. 1c, f, i) epi-illumination light microscopy reveals that the different colors are due to the different optical properties of the scales and the way the scales are arranged on the wing substrate. The wings of *M. epistrophus* are shingled by overlapping rows of pale green-bluish-colored, rather transparent scales; the solid and dotted lines in Fig. 1b, c indicate a cover scale overlapping a ground scale. The wings of *M. helenor* have a quite different scale lattice. Here both cover and ground scales are intensely blue reflecting, however strongly dependent on the direction of illumination and viewing (Fig. 1e, f). The wings of *M. cypris* are even more different. There only ground scales exist, which are highly blue reflecting (Fig. 1h). With oblique illumination the blue reflection is absent, but then it appears that part of the scales contain a brown pigment (Fig. 1i), presumably melanin (Yoshioka and Kinoshita 2006).

### Scale coloration of *M. epistrophus*

**Fig. 2:**
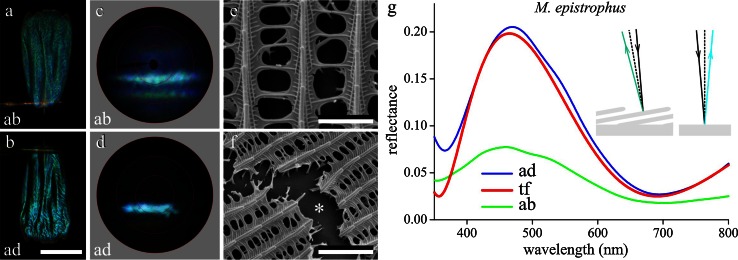
A single scale of *M. epistrophus*. **a**, **b** Micrographs of the *upper* (abwing) and *lower* (adwing) *side*, respectively. **c**, **d** The corresponding scatterograms of a central scale area with diameter ~30 µm. The *red circles* in the scatterograms indicate angles of 5º, 30º, 60º and 90º. **e** Scanning electron micrograph of a scale showing a few ridges and crossribs, framing open windows. **f** Similar micrograph of a damaged scale area, showing the flat lower lamina. **g** Reflectance spectra of both the adwing (*ad*) and abwing (*ab*) side measured with a microspectrophotometer together with the calculated reflectance spectrum of a chitinous thin film (*tf*) with thickness 225 nm (with 0.02 offset); *inset* difference in direction of reflected light by ridge lamellae and lower lamina. *Bars* **a**, **b** 100 µm; **e** 2 µm; **f** 4 µm

To unravel the optical mechanisms causing the wing colorations, we studied the differently colored wing scales by detaching them from the wings and then gluing the single scales to the tip of a glass pipette (Fig. 2a, b). The cover and ground scales of *M. epistrophus* appeared to have a very similar shape and coloration. Applying bright-field epi-illumination microscopy of the slightly wrinkled scales showed that the abwing (upper) side (Fig. 2a) was duller than the adwing side, which was locally rather specular (Fig. 2b). The specularity clearly indicated that the blue color must have a structural basis, and indeed scales immersed in refractive index matching fluid observed with a light microscope in transmission mode were virtually fully transparent (Supplementary Material Fig. S1a, e).

We investigated the spatial distribution of the blue reflections by mounting the scale of Fig. 2a, b in our scatterometer (Stavenga et al. 2009). The scatterogram obtained by illuminating with a narrow-aperture light beam a small spot (diameter ~ 30 µm) at the abwing side showed two main blue bands plus a diffuse blue scattering (Fig. 2c), while the scatterogram of the adwing side featured only one main band (Fig. 2d).

The scatterograms suggested that the structuring of the abwing side of the scales was more complex than that of the adwing side. We therefore performed scanning electron microscopy, which showed that the scales of *M. epistrophus* closely conform with the generic nymphalid scale structure, as shown by Fig. 2e, f (Ghiradella 1998); *cf*. the case of the blue scales of the peacock butterfly, *Aglais io* (Fig. S2; Stavenga et al. 2014). The upper lamina has the classic structure of parallel ridges connected by crossribs, together leaving quite open windows through which the unstructured lower lamina is seen (Fig. 2e). Disruption of the upper lamina showed that the lower lamina is a smooth layer (Fig. 2f).

In other nymphalid butterflies, the lower lamina acts as a thin film (Stavenga et al. 2014), and to check whether that also holds for *M. epistrophus*, we measured reflectance spectra of both scale sides using a microspectrophotometer (Fig. 2g). The reflectance spectrum of the adwing side, with a distinct blue peak very reminiscent of a thin film reflector, well approximated the reflectance spectrum of a chitinous thin film with thickness 225 nm, except for a minor offset (Fig. 2g). Yet, if the lower lamina of the *M. epistrophus* scale indeed acts as a thin film reflector, we might have expected that illumination with a narrow-aperture light beam would have yielded a scatterogram with a distinct, small spot (Stavenga et al. 2009). The broad blue band in the scatterogram of Fig. 2d can be well understood, because the longitudinal wrinkles of the scale will inevitably cause spatial broadening of the reflected beam.

Understanding the scatterogram obtained with abwing illumination (Fig. 2c) requires a more involved explanation. With illumination in the normal way, that is at the abwing side, a part of the incident light passes the ridges and crossribs of the upper lamina and travels through the open windows and then reaches the lower lamina. There predominantly blue light is reflected. The reflected light leaves either again through the windows, thus contributing to the scatterogram a blue-colored band, or it is scattered by the structures of the upper lamina, causing a diffuse blue scattering (Fig. 2c). In addition to entering the windows, some of the incident light hits the ridges and there is partly reflected by the slightly overlapping lamellae. The ridge lamellae sufficiently overlap to let the ridges act as an array of slender multilayers that reflect blue light. This causes a blue-greenish band in the scatterogram, which is spatially shifted with respect to the band created by the lower lamina, because the lamellae are arranged skew to the plane of the lower lamina. The lateral width of the bands is here mainly due to the ridge array acting as a grating (see e.g. Yoshioka and Kinoshita 2004; Stavenga et al. 2009), rather than due to the wrinkled scale surface (Fig. 2c). Actually, a very weak blue-greenish band can be seen in the scatterogram of the adwing side, which is due to light transmitted by the lower lamina and subsequently reflected by the skewed ridge lamellae (Fig. 2d).

Considering now the abwing reflectance spectrum of Fig. 2g, the three components of the abwing reflection mentioned above, i.e., the lower lamina thin film reflection, the ridge lamellae multilayers and the diffuse scattering by the ridge and crossrib structures, together create a slightly undulating reflectance spectrum. The amplitude of the abwing reflectance spectrum is lower than that of the adwing spectrum, because the spatial spread of the abwing reflected and scattered light flux is wider (Fig. 2c), so that the limited aperture of the objective of the microspectrophotometer captures a smaller fraction of the light flux (Fig. 2g).

### Scale coloration of *M. helenor*

**Fig. 3:**
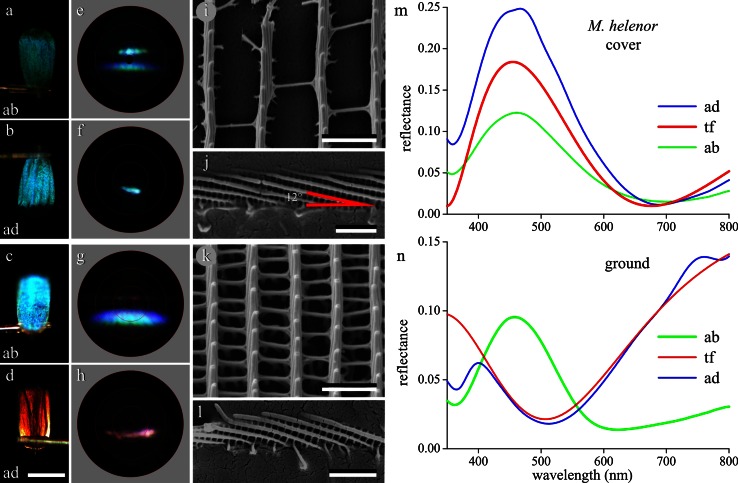
Single cover and ground scales of *M. helenor*. **a**, **b** Micrographs of the cover scale upper (abwing) and lower (adwing) side, respectively. **c**, **d** Micrographs of the ground scale abwing and adwing side, respectively. **e**–**h** Scatterograms of central scale areas corresponding to **a**–**d**. **i** Scanning electron micrograph of a cover scale showing a few very slender ridges and tiny crossribs, framing very large open windows. **j** SEM of a cover scale ridge in side view, showing the stack of overlapping lamellae. **k** SEM of a ground scale with more narrowly spaced ridges connected by numerous crossribs. **l** Side view of a ground scale ridge. **m** Reflectance spectra of both the adwing (*ad*) and abwing (*ab*) side measured with a microspectrophotometer together with the reflectance spectrum calculated for a chitinous thin film (*tf*) with 220 nm thickness. **n** Measured ground scale abwing (*ab*) and adwing (*ad*) reflectance spectra together with the reflectance spectrum for a melanic thin film (*tf*) with 160 nm thickness. *Bars* **a**–**d** 100 µm; **i**, **k** 2 µm; **j**, **l** 1 µm

The cover and ground scales that shingle the wings of *M. helenor* have quite different optical and morphological properties (Fig. 1e, f). Single scales in immersion fluid show that the cover scales are unpigmented (Fig. S1b), while the ground scales contain a distinct amount of melanin-type pigment. Epi-illumination light microscopy of the cover scales shows a slightly lusterless abwing surface (Fig. 3a) and a more specular adwing surface (Fig. 3b). The scatterogram of the abwing side (Fig. 3e) again shows two bands, but less broad than those of Fig. 2c, and the scatterogram of the adwing side features a slightly stretched spot (Fig. 3f). The latter is in full accordance with the approximately flat surface of the lower lamina (Fig. 3b).

The scatterogram of Fig. 3e can be directly related to the morphology revealed by scanning electron microscopy. Figure 3i shows that the cover scale ridges are extremely slender and that they, together with the sparse crossribs, create very large open windows. Alike in the *M. epistrophus* scales, incident light that enters the windows of the *M. helenor* cover scale and subsequently is reflected by the lower lamina creates in the scatterogram an almost spotlike, short blue band, because the lateral spread by the slender ridges is very minor. A second band is created by the ridges, because they consist of stacks of distinctly overlapping lamellae, which thus create a multilayer reflector (Fig. 3j). The two bands of the abwing scatterogram are spatially separated, due to the skewed lamellae (Fig. 3e, j). The angle of the multilayer system with respect to the lower lamina was estimated to be ~12°. The angle between the two bands in the scatterogram was found to be ~25°, as expected for a reflecting multilayer.

The shape of the adwing reflectance spectrum well approximates the spectral shape of a thin film reflector with thickness 220 nm (Fig. 3m). Interestingly, the shapes of the adwing and abwing reflectance spectra of the cover scales are remarkably similar (Fig. 3m), meaning that the reflectance spectra of lower lamina and ridge lamellae are very similar, in agreement with the similar color of the two reflection bands in the abwing scatterogram (Fig. 3e). Apparently the lower lamina thin film thickness and ridge multilayer spacings are well-tuned, creating together a saturated blue color.

Observing the ground scales with the epi-illumination microscope at the abwing side reveals again a bright blue reflection (Fig. 3c), but the adwing side is locally specular with a red-brown-purplish color (Fig. 3d). The abwing scatterogram shows a main, bright blue band together with a faint red band (Fig. 3g), but in the adwing scatterogram a very different red-purplish band is seen (Fig. 3h). These patterns can be readily explained by the scale morphology. The ridges of the ground scales form a rather dense array consisting of stacked lamellae (Fig. 3k, l), responsible for the prominent blue band in the abwing scatterogram (Fig. 3g).

This is quite in accordance with the abwing reflectance spectrum, which has a clear band peaking in the blue wavelength region. Its bandwidth is smaller than that of a thin film, characteristic for a multilayer (Kinoshita et al. 2002a). The adwing reflectance spectrum with a trough around 500 nm is well approximated by the reflectance spectrum of a melanic thin film (tf in Fig. 3n) with thickness 160 nm. The lower lamina acting as a thin film reflector thus causes the clear purplish band in the adwing scatterogram and as well the faint band in the abwing scatterogram. Actually, ground scales observed in transmitted light show that they are heavily pigmented with a melanin-like pigment. This was incorporated in the calculations of the tf-spectrum of Fig. 3n (see “Materials and methods”).

### Scale coloration of *M. cypris*

**Fig. 4:**
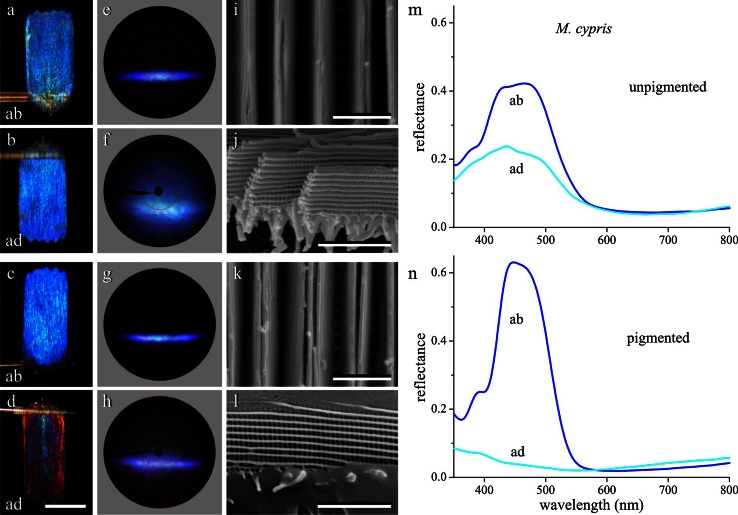
An unpigmented and pigmented scale of *M. cypris*. **a**, **b** Micrographs of an unpigmented single scale, abwing (*ab*) and adwing (*ad*). **c**, **d** Micrographs of a pigmented single scale. **e**, **f** Scatterograms of the unpigmented scale. **g**, **h** Scatterograms of the pigmented scale. **i**, **k** SEM of the upper lamina in normal view of an unpigmented and pigmented scale, respectively. **j**, **l** Lateral view of the large stack of scale lamellae. **m**, **n** MSP reflectance spectra of the *ab*- and *ad*-wing sides of an unpigmented and pigmented scale, respectively. *Bars* **a**–**d** 100 µm; **i**–**l** 2 µm

The wings of *M. cypris* have two main types of scales, unpigmented and pigmented (Figs. 1i, 4). Upon normal epi-illumination of the abwing side, both scale types are strikingly blue (Fig. 4a, c). The adwing side of the unpigmented scale is similarly blue reflecting (Fig. 4b), but the adwing side of the pigmented scale is blue to brown, depending on the location in the scale (Fig. 4d). The corresponding scatterograms all show a prominent blue band, clearly due to the dense array of parallel, tall ridges (Fig. 4i–k). The ridges consist of a high stack of lamellae, creating a strongly reflecting multilayer that causes narrow-band reflectance spectra (Fig. 4m, n). Compared to the ridge multilayers, the reflection by the lower lamina is apparently negligible, because the abwing scatterograms only feature a single band, unquestionably arising from the ridges. The adwing scatterograms show in addition to the reflection band a slightly diffuse pattern. The latter is presumably due to light transmitted by the lower lamina which then is reflected by the ridges and in addition somewhat diffused by the lower parts of the ridges.

## Discussion and conclusion

The dorsal wings of many butterflies of the genus *Morpho* are blue colored. By comparing a few characteristic *Morpho* species, we have found that the lower lamina of the scales acting as a thin reflector can exclusively cause the blue coloration, but more intense blue reflections result when the scale ridges have tall stacks of lamellae, acting as highly reflective multilayers. Increasing the number of lamellae layers and decreasing the distance between the ridges enhance the scale reflectance and so the butterfly’s brightness.

Expression of the broadband absorbing pigment melanin is an effective method to reduce straylight and backscattering and so to prevent the decrease in saturation of the color. This is immediately clear from the case of *M. epistrophus*, where the virtual absence of melanin results in an unsaturated blue color. Although cover and ground scales can individually be distinctly blue (Fig. 2), overlapping cover and ground scales desaturate the blue color, as is also the case in nymphaline butterflies (Stavenga et al. 2014). Saturated, bright-blue colors result with ample melanin pigmentation of the ground scales of *M. helenor* and *M. cypris*.

Not only the architecture of the scales of the different *Morpho* species but also their arrangement on the wings highly varies (Kinoshita 2008). In *M. epistrophus* cover and ground scales are extremely similar and their structure is almost identical to those of the little developed scales of nymphaline butterflies (Stavenga et al. 2014). Cover and ground scales of *M. helenor* are structured very differently, however. Whereas the cover scales have minor ridges with multilayers leaving the lower lamina well-exposed, the ground scales have prominent multilayered ridges upon a pigmented lower lamina. The overlapping scales on the wing together determine the wing coloration (see Fig. S3). A very similar arrangement as that of *M. helenor* is encountered in *M. didius*. On the wing, the cover scales are thought to act as diffusers for the light reflected by the ground scales (Vukusic et al. 1999; Yoshioka and Kinoshita 2004).

In *M. cypris* the cover scales are absent, but dependent on the location the scales as well as the wing substrate are pigmented, which causes the banded color of the wings (Yoshioka and Kinoshita 2006). The absence of cover scales means that the scale reflections are spatially more directional than in *M. helenor*. In *M. cypris* the directional signaling is weakened by the large white areas of the wing. Other species, like *M. aega*, seem to have more perfectioned spatial signaling as their wings are virtually fully covered by highly reflective and melanin-pigmented ground scales, which enables them to send out very directional visual signals.

The three species we have studied demonstrate that the coloration of *Morpho* butterflies is highly variable, presumably as a consequence of a strong evolutionary process. A further comparative study on other Morphinae will reveal whether scale and wing coloration indeed parallels *Morpho* evolution.

## Electronic supplementary material

Below is the link to the electronic supplementary material.

**Fig. S1** Pigmentation of *Morpho* scales. *M.e*., *M. epistrophus*; *M.h.*, *M. helenor*; *M.c.*, *M. cypris*. (**a**-**d**) Micrographs of scales immersed in oil (*n* = 1.515) of *M. epistrophus* (**a**), *M. helenor* (cover, **b**), *M. cypris* (unpigmented, **c**) and *M. cypris* (pigmented, **d**). (**e**) Absorbance spectra measured with a microspectrophotometer of *M. epistrophus*, *M. helenor* (cover and ground) and *M. cypris* (unpigmented and pigmented) together with the absorbance spectrum calculated for a melanin-pigmented scale with thickness 160 nm and imaginary part of the refractive index given by 2.5exp(-*λ*/270), with wavelength *λ* in nm (JPEG 196 kb)

**Fig. S2** Blue scales of the peacock *Aglais io*. **a**, **b** Micrographs of the abwing (ab) and adwing (ad) sides of a single blue scale. **c**, **d** Scatterograms of a small area of **a** and **b**. **e** Scanning electron micrograph of the scale with ridges and crossribs. **f** Scanning electron micrograph of a scale with a torn upper lamina showing the smooth, flat lower lamina and the trabeculae that join both laminae. **g** Reflectance spectra of the adwing and abwing sides of the scale pictured in **a** and **b**; inset: the right wings of the peacock, showing the large patch with blue scales. Bars: **a**, **b** 50 µm; **e** 2 µm; **f** 5 µm (JPEG 330 kb)

**Fig. S3** Optics of a locally damaged wing of a *Morpho helenor*. **a** 1: Cover scale overlapping a ground scale and wing substrate (cgs); 2: cover scale without a ground scale on substrate (cs); 3: ground scale on substrate (gs); 4: only substrate (s). **b**-**e** Scatterograms of locations 1-4. 1: the two diffraction bands due to cover and ground scales are a combination of Fig. 3e and 3 g, showing that cover and ground scales are about parallel, since the bottom pattern of cover scale and the one of the ground scale fuse; 2: An additional brown band, due to the reflecting wing substrate, is displaced from the bands due to the cover scale, showing that the cover scale is inclined to the wing substrate; 3: The blue band from the ground scale and the brown band due to the substrate are well displaced; 4: The wing substrate only acts as a non-ideal thin reflector. **f** Reflectance spectra from locations 1-4 measured with a microspectrophotometer. 1, 2: Spectra of thin film reflectors, as Fig. 3 m; 3: Spectrum as that of the abwing scale reflectance spectrum of Fig. 3n; 4: Two reflectance spectra of slightly different locations of the wing substrate, showing characteristic thin film oscillation, indicating a wing thickness of ~ 0.8 µm. Bar: **a** 100 µm (JPEG 239 kb)
