# Supplementary figures and images for: Brilliant iridescence of Morpho butterfly wing scales is due to both a thin film lower lamina and a multilayered upper lamina
Source: J Comp Physiol A Neuroethol Sens Neural Behav Physiol. 2016 Apr 12;202:381–8. doi: 10.1007/s00359-016-1084-1 (PMC4841846; doi:10.1007/s00359-016-1084-1)

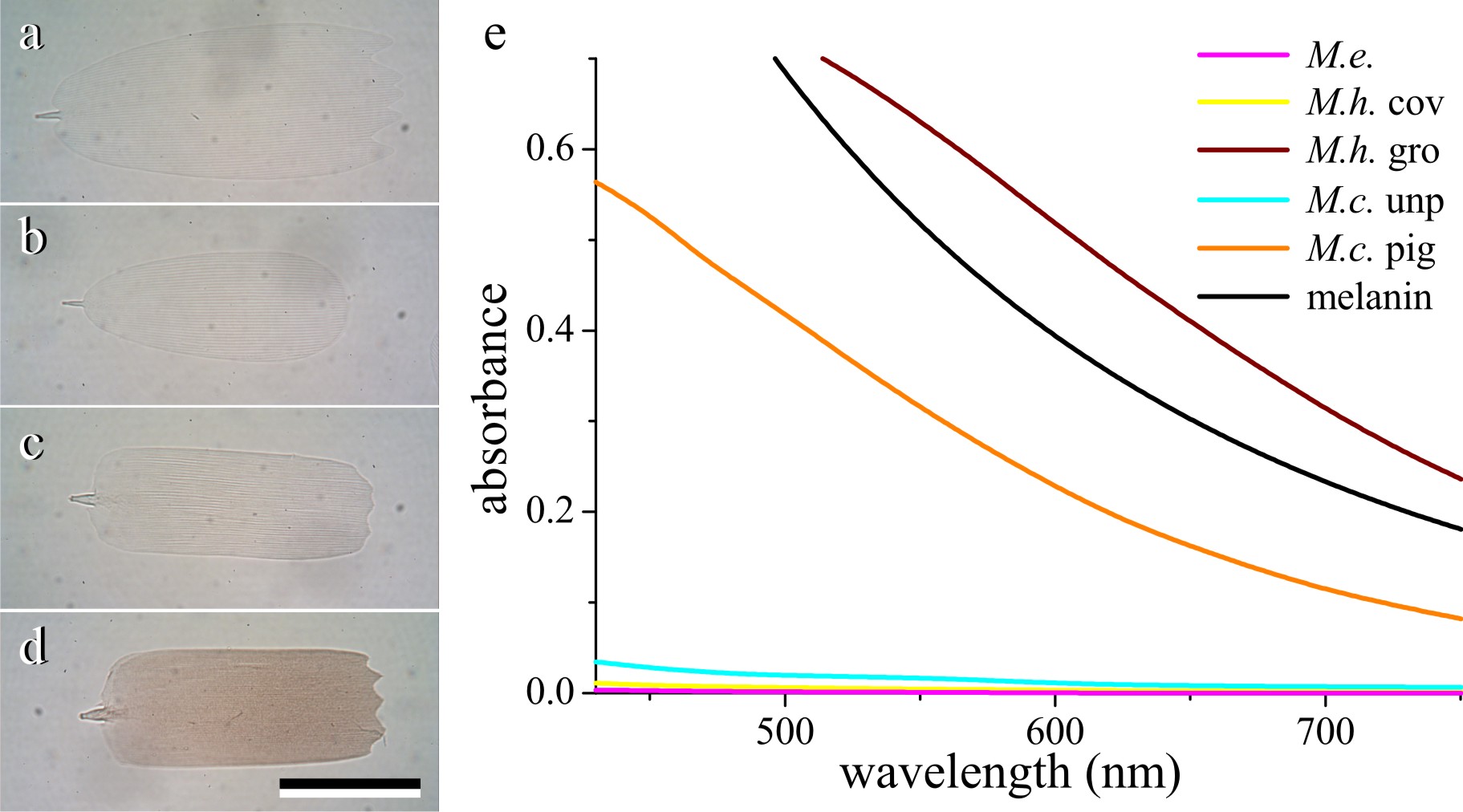

Supplement: Supplementary file 1 — Fig. S1 Pigmentation of Morpho scales. M.e., M. epistrophus; M.h., M. helenor; M.c., M. cypris. (a-d) Micrographs of scales immersed in oil (n = 1.515) of M. epistrophus (a), M. helenor (cover, b), M. cypris (unpigmented, c) and M. cypris (pigmented, d). (e) Absorbance spectra measured with a microspectrophotometer of M. epistrophus, M. helenor (cover and ground) and M. cypris (unpigmented and pigmented) together with the absorbance spectrum calculated for a melanin-pigmented scale with thickness 160 nm and imaginary part of the refractive index given by 2.5exp(-λ/270), with wavelength λ in nm (JPEG 196 kb) [file 359_2016_1084_MOESM1_ESM.jpg]

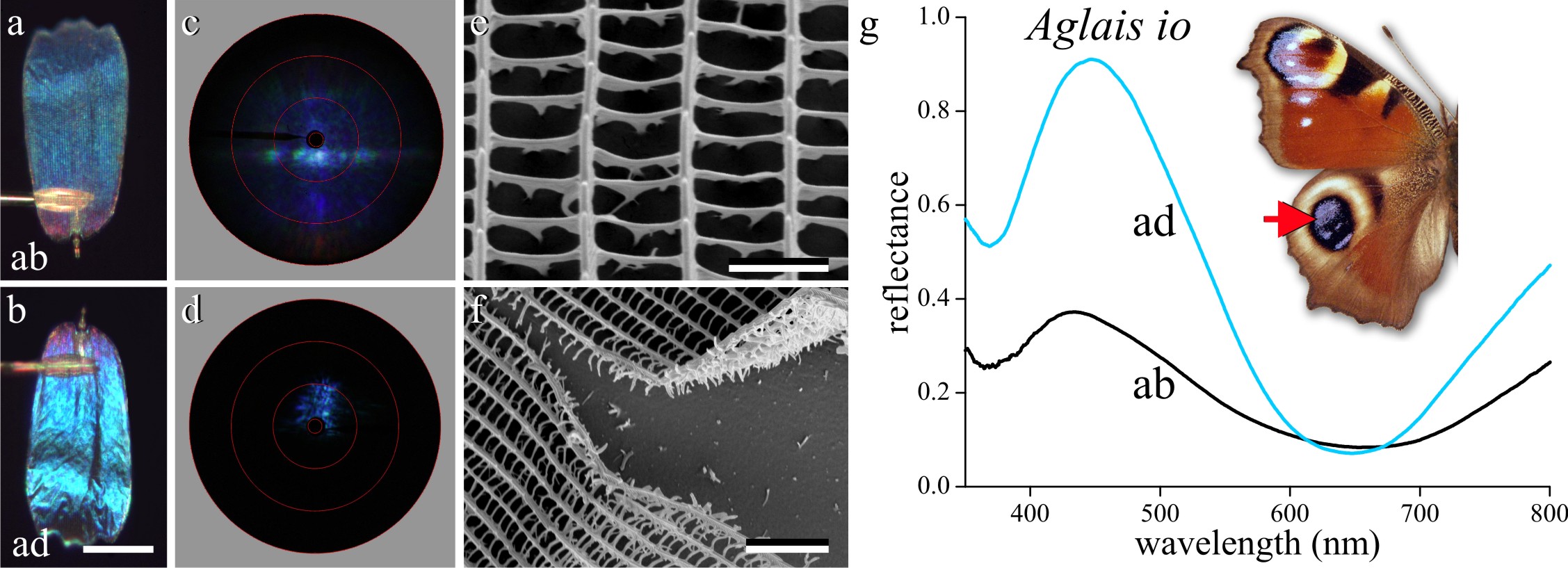

Supplement: Supplementary file 2 — Fig. S2 Blue scales of the peacock Aglais io. a, b Micrographs of the abwing (ab) and adwing (ad) sides of a single blue scale. c, d Scatterograms of a small area of a and b. e Scanning electron micrograph of the scale with ridges and crossribs. f Scanning electron micrograph of a scale with a torn upper lamina showing the smooth, flat lower lamina and the trabeculae that join both laminae. g Reflectance spectra of the adwing and abwing sides of the scale pictured in a and b; inset: the right wings of the peacock, showing the large patch with blue scales. Bars: a, b 50 µm; e 2 µm; f 5 µm (JPEG 330 kb) [file 359_2016_1084_MOESM2_ESM.jpg]

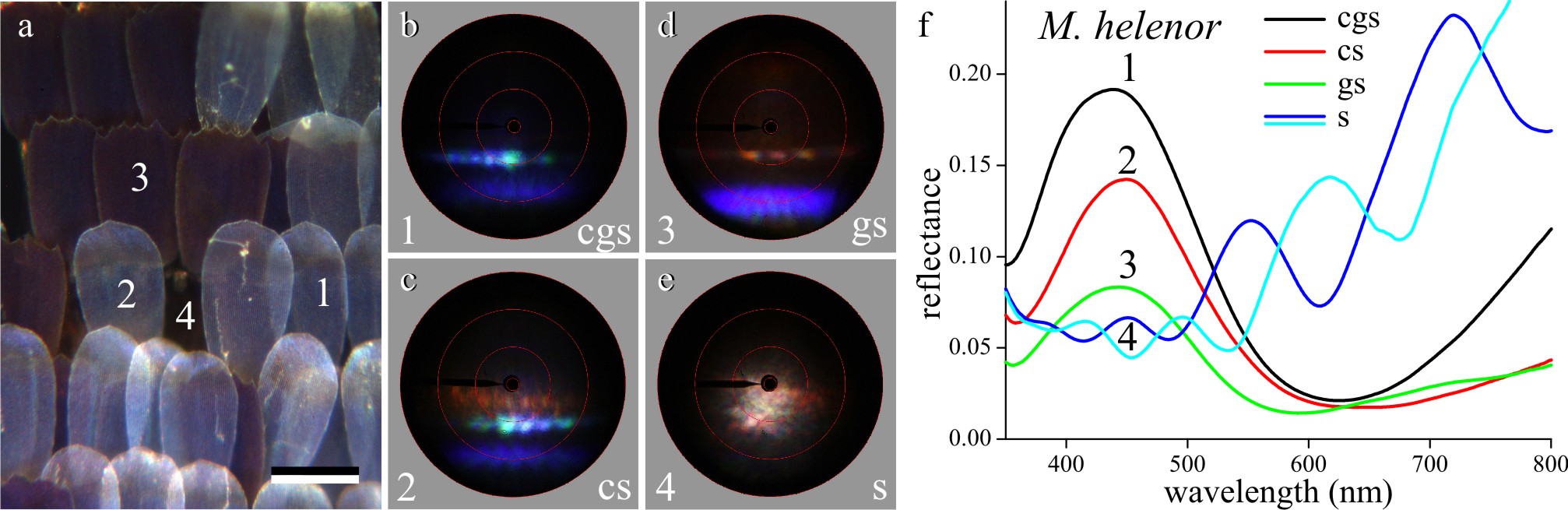

Supplement: Supplementary file 3 — Fig. S3 Optics of a locally damaged wing of a Morpho helenor. a 1: Cover scale overlapping a ground scale and wing substrate (cgs); 2: cover scale without a ground scale on substrate (cs); 3: ground scale on substrate (gs); 4: only substrate (s). b-e Scatterograms of locations 1-4. 1: the two diffraction bands due to cover and ground scales are a combination of Fig. 3e and 3 g, showing that cover and ground scales are about parallel, since the bottom pattern of cover scale and the one of the ground scale fuse; 2: An additional brown band, due to the reflecting wing substrate, is displaced from the bands due to the cover scale, showing that the cover scale is inclined to the wing substrate; 3: The blue band from the ground scale and the brown band due to the substrate are well displaced; 4: The wing substrate only acts as a non-ideal thin reflector. f Reflectance spectra from locations 1-4 measured with a microspectrophotometer. 1, 2: Spectra of thin film reflectors, as Fig. 3 m; 3: Spectrum as that of the abwing scale reflectance spectrum of Fig. 3n; 4: Two reflectance spectra of slightly different locations of the wing substrate, showing characteristic thin film oscillation, indicating a wing thickness of ~ 0.8 µm. Bar: a 100 µm (JPEG 239 kb) [file 359_2016_1084_MOESM3_ESM.jpg]
